# B6 Donor Lungs Develop Chronic Lung Allograft Dysfunction–like Pathology Across Histocompatibility Mismatches in Murine Lung Transplantation

**DOI:** 10.1097/TXD.0000000000001958

**Published:** 2026-05-21

**Authors:** Taisuke Kaiho, Yudai Miyashita, Taichi Nagano, Raul Piseaux Aillon, Yuriko Yagi, Takahide Toyoda, G.R. Scott Budinger, Ankit Bharat, Hidemi Suzuki, Chitaru Kurihara

**Affiliations:** 1 Division of Thoracic Surgery, Department of Surgery, Northwestern University Feinberg School of Medicine, Chicago, IL.; 2 Division of Pulmonary and Critical Care, Department of Medicine, Northwestern University Feinberg School of Medicine, Chicago, IL.; 3 Department of General Thoracic Surgery, Chiba University Graduate School of Medicine, 1Chiba, Japan.

## Abstract

**Background.:**

Chronic lung allograft dysfunction (CLAD) remains a major barrier to long-term survival after lung transplantation. Murine models are essential for mechanistic studies, but the influence of donor-recipient strain direction and histocompatibility mismatch on chronic pathology is not fully defined. We evaluated the reproducibility of B6-background donor lungs across reciprocal strain combinations as a practical experimental platform.

**Methods.:**

Orthotopic left lung transplantation was performed using major-mismatched (C57BL/6 [B6]↔BALB/c) and minor-mismatched (B6↔C57BL/10 [B10]) combinations. Major-mismatched recipients received costimulatory blockades (anti-CD40L/cytotoxic T-lymphocyte–associated protein 4 Ig); minor-mismatched pairs received no immunosuppression. Grafts were procured on day 28. Chronic injury was quantified using standardized histological scoring for airway, parenchymal, and pleural compartments. Lung function was assessed using the FlexiVent system with right hilar clamping.

**Results.:**

CLAD-like airway and fibrotic lesions developed across all combinations, irrespective of donor-recipient direction. B6 donor lungs reproducibly exhibited chronic pathology comparable with other strains. Notably, the incidence of obliterative bronchiolitis was higher in minor-mismatched groups (B10→B6: 54.5%; B6→B10: 38.5%) compared with major-mismatched groups (BALB/c→B6: 27.3%; B6→BALB/c: 36.4%). Histological severity significantly correlated with impaired lung compliance and increased airflow resistance, supporting the functional relevance of the observed structural injury.

**Conclusions.:**

Reciprocal murine lung transplantation reveals that CLAD-like pathology develops irrespective of donor-recipient direction, with mismatch degree influencing the qualitative distribution of chronic injury. These findings validate B6-background donor lungs as a versatile platform for interrogating donor-intrinsic immune and stromal mechanisms of chronic rejection, and for hypothesis-driven testing of donor-targeted interventions using genetically modified strains.

## Graphical abstract

**Figure:**
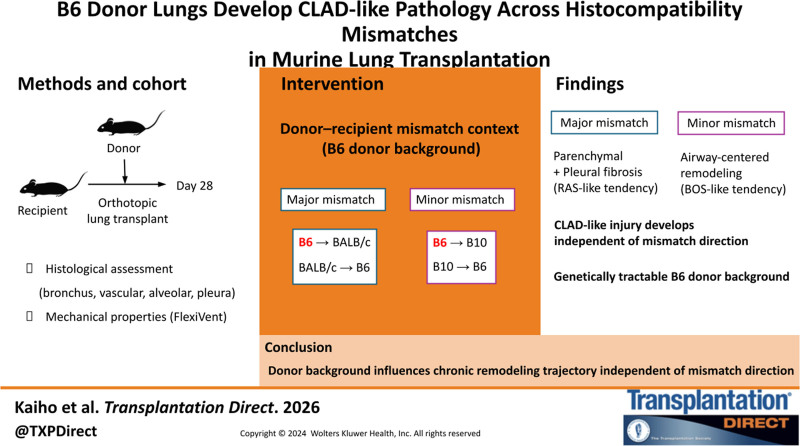

Chronic lung allograft dysfunction (CLAD), which includes bronchiolitis obliterans syndrome (BOS) and restrictive allograft syndrome (RAS), remains the leading cause of late graft failure after lung transplantation.^1^ Despite advances in perioperative management and immunosuppression, no therapy reliably prevents the progressive inflammation and fibrosis that define CLAD, underscoring persistent gaps in our understanding of the biological determinants of chronic rejection. In murine CLAD models, heterotopic and orthotopic tracheal allograft models,^2^ as well as orthotopic lung transplantation, have been internationally recognized as complementary experimental systems to study airway injury, alloimmune responses, and fibrotic remodeling.^3^ Among these, orthotopic murine lung transplantation was first established in 2007 as a reproducible in vivo model of lung allograft injury and chronic rejection.^4^

Prior studies have demonstrated that donor-recipient histocompatibility mismatch influences the spectrum of chronic rejection phenotypes in murine lung allografts.^5^ Several orthotopic lung transplant studies have demonstrated that specific strain combinations, including C57BL/6 (B6), BALB/c, and C57BL/10 (B10), reproducibly develop obliterative airway pathology resembling CLAD.^4,6-10^ In most prior reports, however, B6 mice have predominantly been used as recipients, reflecting their widespread use as the genetic backbone for engineered mouse strains. In contrast, the behavior of B6 lungs as donors, particularly across reciprocal donor-recipient directions and distinct degrees of histocompatibility mismatch, has been less systematically examined. As a result, how donor strain identity and transplantation direction influence the distribution and severity of chronic lung allograft injury remains incompletely defined.

To address this gap, we performed reciprocal orthotopic left lung transplantation using B6, BALB/c, and B10 mice to compare chronic rejection phenotypes across major and minor histocompatibility mismatches. Using a modified cuff technique as previously described,^6^ we generated 4 donor-recipient combinations representing major and minor histocompatibility mismatches: BALB/c→B6, B6→BALB/c, B10→B6, and B6→B10. To prevent acute rejection in major-mismatched transplants, recipients received perioperative immunosuppression with anti-CD40L (MR1; 250 μg, intraperitoneally on day 0) and cytotoxic T-lymphocyte–associated protein 4 Ig (200 μg, intraperitoneally on day 2), administered at doses previously reported to permit long-term graft survival in murine lung transplantation models.^4^ Minor-mismatched B6↔B10 pairs underwent transplantation without immunosuppression. Grafts were procured on postoperative day 28. All animal experiments were approved by the Institutional Animal Care and Use Committee of Northwestern University and were conducted in accordance with the National Institutes of Health Guide for the Care and Use of Laboratory Animals.

Mechanical properties of the transplanted lung were assessed using the FlexiVent system.^11^ Measurements were performed under open-chest conditions, with clamping of the right pulmonary hilum to minimize the contribution of the native right lung and mediastinal structures. Although this approach permits preferential assessment of the transplanted left lung, we acknowledge that compensatory mechanics from residual thoracic structures cannot be fully excluded, and lung function data were therefore interpreted in conjunction with histologic findings.

Histologic assessment was performed on formalin-fixed, paraffin-embedded sections stained with hematoxylin and eosin and Masson’s trichrome. Chronic rejection was evaluated using International Society for Heart and Lung Transplantation A and B scores for perivascular and airway inflammation,^12^ the modified Ashcroft scale for parenchymal fibrosis,^13^ and additional metrics quantifying obliterative airway fibrosis,^7^ peribronchiolar thickness, and pleural fibrosis. Perivascular lymphoid aggregates were distinguished from acute vascular rejection during A scoring, consistent with prior descriptions of organized lymphoid structures in immunomodulated murine lung transplants.^14^ These approaches enabled comprehensive assessment across bronchial, vascular, alveolar, and pleural compartments.

A total of 11–13 mice were analyzed in each donor-recipient group. Representative histologic sections (Figure 1A) demonstrated CLAD-like pathology across all donor-recipient combinations, including those with B6 donors, with involvement of multiple anatomic compartments, including airway narrowing or obliteration, peribronchial and perivascular inflammatory infiltrates, alveolar fibrotic remodeling, and pleural thickening. These findings indicate that B6 donor lungs, similar to BALB/c and B10 donors, are capable of sustaining chronic allograft injury in orthotopic lung transplantation.

**FIGURE 1.**
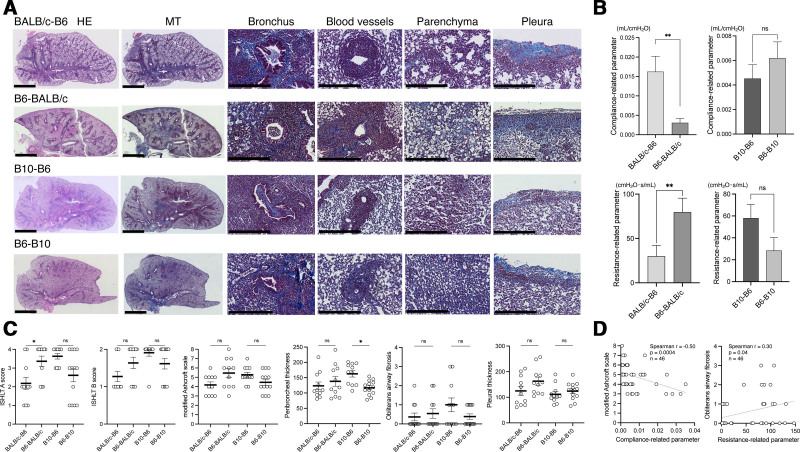
Donor strain–dependent patterns of CLAD in murine orthotopic lung transplantation. A, Representative histologic images of transplanted lungs from each donor-recipient combination at postoperative day 28. Low-magnification views with H&E and MT staining, together with high-magnification MT images, illustrate CLAD-like pathology across multiple anatomic compartments, including bronchiolar lesions with airway narrowing or obliteration, perivascular inflammatory infiltrates, parenchymal (alveolar) fibrotic remodeling, and pleural thickening. Scale bars: 2.5 mm (H&E and MT whole-lung images) and 250 μm (higher-magnification images). B, Mechanical properties of the transplanted lungs assessed using the FlexiVent system under open-chest conditions with right pulmonary hilar clamping. Compliance-related and resistance-related parameters were measured selectively in the left transplanted lung. Statistical significance was determined using the Mann-Whitney *U* test for 2-group comparisons. C, Quantitative histopathologic assessment of transplanted lungs using standardized scoring systems, including ISHLT A and B scores, the modified Ashcroft scale, peribronchiolar thickness, pleural thickness, and obliterative airway fibrosis scores. Obliterated airways were graded as follows: 0 = 0%, 1 = 1%–25%, 2 = 26%–50%, 3 = 51%–75%, and 4 = 76%–100% luminal obliteration. Data represent n = 11–13 mice per group. Statistical significance was determined using 1-way ANOVA for comparisons among multiple groups. D, Correlation between compliance–related parameters and parenchymal fibrosis assessed by the modified Ashcroft scale, and between resistance-related parameters and obliterative airway fibrosis scores, analyzed using Spearman’s rank correlation. Regression lines are shown for visualization purposes only. CLAD, chronic lung allograft dysfunction; H&E, hematoxylin and eosin; ISHLT, International Society for Heart and Lung Transplantation; MT, Masson’s trichrome.

Functional assessment of the transplanted lungs is summarized in Figure 1B. In major-mismatched transplants, the B6→BALB/c group exhibited significantly greater impairment in compliance-related and resistance-related parameters compared with the BALB/c→B6 group. In contrast, under minor-mismatched conditions, the B10→B6 group demonstrated lower respiratory system compliance–related parameters and higher resistance-related parameters than the B6→B10 group. These findings suggest that donor strain identity and transplantation direction are associated with distinct patterns of functional decline, although functional differences were modest and interpreted in the context of histologic injury.

Histopathologic scoring revealed donor-dependent trends consistent with these functional observations (Figure 1C). Across all donor-recipient combinations, lung transplantation from B6 donors consistently resulted in the development of CLAD-like lesions. While quantitative differences in severity were observed among groups, the overall presence of chronic rejection–associated pathology was reproducible and comparable. When stratified by the degree of histocompatibility mismatch, differences in the anatomic distribution of chronic rejection became apparent. In major-mismatched transplants, grafts from B6 donors exhibited relatively greater involvement of alveolar and pleural compartments, as reflected by higher modified Ashcroft scores and increased pleural thickness. In contrast, under minor-mismatched conditions, particularly in grafts transplanted into B6 recipients, airway-centered lesions, including peribronchiolar fibrosis and obliterative airway changes, were more prominent. Consistent with this airway-predominant pattern, the incidence of obliterative bronchiolitis was higher in minor-mismatched groups (B10→B6, 54.5%; B6→B10, 38.5%) than in major-mismatched groups (BALB/c→B6, 27.3%; B6→BALB/c, 36.4%; Table 1). Correlation analyses revealed an inverse association between compliance–related parameters and the modified Ashcroft scale, and a positive association between resistance-related parameters and obliterative airway fibrosis severity (Figure 1D).

**Table 1.** Experimental groups and incidence of obliterative bronchiolitis in murine orthotopic lung transplantation

Experimental group	BALB/c-B6	B6-BALB/c	B10-B6	B6-B10
Donor	BALB/c	C57BL/6J	C57BL/10J	C57BL/6J
Recipient	C57BL/6J	BALB/c	C57BL/6J	C57BL/10J
Immunosuppressant	Yes	Yes	No	No
Obliterative bronchiolitis/total mice, n/N (%)	3/11 (27.3%)	4/11 (36.4%)	6/11 (54.5%)	5/13 (38.5%)

B6, C57BL/6J; B10, C57BL/10J.

These findings indicate that although B6 donor lungs reliably develop CLAD-like pathology irrespective of mismatch degree, the qualitative distribution of chronic injury differs according to donor-recipient context. Although the present study was not powered to define discrete phenotypes, the compartment-specific distribution of chronic lesions differed according to histocompatibility mismatch. Major histocompatibility mismatch was associated with relatively greater parenchymal and pleural involvement, whereas minor histocompatibility mismatch preferentially exhibited airway-centered remodeling. These qualitative patterns are conceptually consistent with RAS-like and BOS-like remodeling tendencies, respectively, but should be interpreted as trends rather than definitive phenotypic assignments.

CLAD development in clinical lung transplantation is not strictly dependent on the magnitude of minor histocompatibility complex mismatch alone. The lung is uniquely exposed to environmental stimuli and is enriched in innate immune populations—including neutrophils, monocytes, macrophages, and innate lymphoid cells—that can propagate chronic inflammation and remodeling independent of adaptive alloimmune intensity. Activation of pattern-recognition receptors and danger-associated molecular pathways following epithelial injury may contribute to sustained airway or parenchymal fibrosis even in the absence of strong histocompatibility disparities. In this context, the mismatch-dependent compartmentalization observed in our model may reflect differential engagement of innate inflammatory circuits in addition to adaptive alloimmune mechanisms.

Importantly, human CLAD is characterized by airway-centered fibroproliferative remodeling in BOS and parenchymal and pleural fibrosis in RAS, accompanied by persistent immune-epithelial-stromal interactions. Recent comprehensive reviews have emphasized that CLAD represents a clinically defined syndrome encompassing heterogeneous immunologic and nonimmunologic drivers, including infection, innate immune activation, and alloantibody responses.^15^ The compartment-specific injury patterns observed in this murine platform align with these core structural features described in human CLAD, suggesting that conserved biological programs may underlie chronic lung allograft injury across species. Although murine models cannot fully recapitulate the clinical heterogeneity of human CLAD, they provide a tractable system in which shared immune and tissue remodeling pathways can be mechanistically interrogated.

Notably, epithelial alterations such as club cell loss, a recognized feature of CLAD in both murine^16^ and human lungs,^17^ were not directly assessed in this study and warrant focused investigation in future work. In support of this concept, studies in murine kidney transplantation have demonstrated that even fully mismatched strain combinations can display markedly different rejection kinetics depending on donor-recipient pairing.^18^ These observations suggest that donor-derived factors critically influence graft fate across organ systems. Importantly, clinical studies have demonstrated that CLAD may occur even when donor-specific antibodies are crossed without uniformly adverse long-term outcomes, indicating that the presence or magnitude of adaptive alloimmune mismatch alone does not dictate chronic graft.^19^ Together, these findings reinforce the concept that graft-intrinsic programs and innate immune mechanisms contribute substantially to CLAD pathogenesis.

The predominance of B6 mice as the genetic background for engineered models underscores the relevance of these findings. Demonstrating that B6 donor lungs reproducibly develop CLAD-like injury across mismatch contexts establishes a practical experimental platform in which genetically modified donor lungs can be interrogated without recipient-targeted interventions that may confound interpretation. This concept aligns with emerging evidence that donor lung–intrinsic immune cell crosstalk has been shown to shape early alloimmune injury, emphasizing the capacity of graft-resident programs to influence rejection outcomes.^20^

Because the majority of genetically engineered mouse strains are generated on a C57BL/6 background, this B6 donor–based platform uniquely enables donor-intrinsic interrogation of CLAD biology. By selectively manipulating donor-derived immune, epithelial, or stromal pathways while maintaining controlled recipient conditions, this system permits hypothesis-driven testing of mechanisms regulating airway obliteration, parenchymal fibrosis, and pleural remodeling. The mismatch-dependent compartmentalization of chronic injury observed here supports the testable hypothesis that donor-intrinsic programs may differentially shape airway- versus parenchyma-predominant remodeling trajectories. This conceptual framework is consistent with recent discussions in transplantation emphasizing the integration of mechanistic immune profiling, molecular characterization, and clinically defined CLAD phenotypes.^21^

Furthermore, this framework allows prospective evaluation of donor-targeted strategies—including genetic perturbation or ex vivo modulation of donor lungs before implantation—to determine whether early donor-resident immune and tissue programs influence the later development of CLAD-like pathology. In this manner, the present study extends beyond a descriptive model and establishes a biologically actionable platform for mechanistic and interventional investigations.

A key consideration in interpreting this model is that CLAD-like remodeling developed in the absence of maintenance immunosuppression. Accordingly, this system is not intended to fully recapitulate the clinical setting of lung transplantation under contemporary immunosuppressive regimens. Rather, it provides an experimentally tractable platform to examine how donor-recipient histocompatibility mismatch shapes chronic airway-centered allograft injury in vivo. Within this framework, the model may be particularly useful for mechanistic studies aimed at dissecting alloimmune pathways that promote CLAD-like remodeling and may serve as a foundation for future interventional studies incorporating immunosuppressive agents, costimulatory blockade, or other targeted strategies to test approaches that prevent chronic graft deterioration.

This study has several limitations. First, a no-mismatch isograft control group was not included. Because the aim of this study was to compare CLAD-like pathology across distinct mismatch settings, we focused on major and minor histocompatibility disparities. Nonetheless, inclusion of isograft controls in future studies will be important to define the baseline extent of chronic remodeling unrelated to alloimmune mismatch and to further contextualize the effects observed here.

Second, although our analyses focused on global histological remodeling and functional decline, specific epithelial alterations such as club cell loss—a recognized feature of human CLAD—were not directly assessed. Third, although right hilar clamping was used during physiological measurements to preferentially assess the transplanted lung, compensatory mechanical contributions from residual thoracic structures cannot be entirely excluded. Finally, this study was designed to evaluate the reproducibility of a B6 donor–based experimental platform rather than to define discrete CLAD phenotypes; therefore, the observed patterns should be interpreted as qualitative trends rather than definitive phenotypic assignments. Future studies incorporating cell-specific analyses and longitudinal assessments will further refine the applicability of this model.

In conclusion, reciprocal murine orthotopic lung transplantation demonstrates that CLAD-like pathology develops across both major and minor histocompatibility mismatches irrespective of donor-recipient direction. The impact of B6 donor lungs on chronic rejection differs between major and minor histocompatibility mismatch settings, resulting in distinct patterns of chronic injury that are conceptually consistent with RAS-like and BOS-like remodeling tendencies. These findings support the use of B6-background donor lungs as a versatile experimental platform for mechanistic studies of CLAD and donor-intrinsic determinants of chronic graft failure.

## ACKNOWLEDGMENTS

Comparative histopathology and molecular phenotyping services were provided by the Northwestern University Mouse Histology and Phenotyping Laboratory (RRID:SCR_017870), which is supported by the National Cancer Institute (P30-CA060553) awarded to the Robert H. Lurie Comprehensive Cancer Center.
